# A pooled analysis of outcomes according to cytogenetic abnormalities in patients receiving ixazomib- vs placebo-based therapy for multiple myeloma

**DOI:** 10.1038/s41408-022-00768-5

**Published:** 2023-01-12

**Authors:** Wee-Joo Chng, Sagar Lonial, Gareth J. Morgan, Shinsuke Iida, Philippe Moreau, Shaji K. Kumar, Philip Twumasi-Ankrah, Miguel Villarreal, Ajeeta B. Dash, Alexander Vorog, Xiaoquan Zhang, Kaveri Suryanarayan, Richard Labotka, Meletios A. Dimopoulos, S. Vincent Rajkumar

**Affiliations:** 1 Department of Hematology-Oncology, National University Cancer Institute, Singapore, Singapore; 2 Cancer Science Institute of Singapore, National University of Singapore, Singapore, Singapore; 3 Department of Hematology and Medical Oncology, Winship Cancer Institute, Emory University Medical School, Emory University, Atlanta, GA USA; 4 Perlmutter Cancer Center, NYU Langone Health, New York, NY USA; 5 Department of Hematology and Oncology, Nagoya City University Institute of Medical and Pharmaceutical Sciences, Nagoya, Japan; 6 Hematology Department, University Hospital Hotel Dieu, Nantes, France; 7 Division of Hematology, Department of Internal Medicine, Mayo Clinic, Rochester, MN USA; 8 Takeda Development Center Americas, Inc. (TDCA), Lexington, MA USA; 9 Hematology and Medical Oncology, Department of Clinical Therapeutics, National and Kapodistrian University of Athens, School of Medicine, Athens, Greece

**Keywords:** Myeloma, Cytogenetics, Myeloma, Myeloma, Phase III trials

## Abstract

Some cytogenetic abnormalities (CAs) are associated with poorer prognosis in multiple myeloma (MM); proteasome inhibitors appear to benefit patients with high-risk CAs. We evaluated 2247 MM patients from the TOURMALINE-MM1/-MM2/-MM3/-MM4 trials to assess the PFS benefit of ixazomib plus lenalidomide-dexamethasone (Rd) vs placebo-Rd (TOURMALINE-MM1/-MM2) or ixazomib vs placebo (TOURMALINE-MM3/-MM4) in specific high-risk CAs. After a pooled median follow-up of 25.6 months, the hazard ratio (HR) for PFS with ixazomib- vs placebo-based therapy for high-risk patients was 0.74 (95% confidence interval [CI]: 0.59–0.93; median PFS [mPFS] 17.8 vs 13.2 months), and 0.70 (95% CI: 0.62–0.80; mPFS 26.3 vs 17.6 months) for complementary standard-risk patients. The HR for expanded high-risk patients was 0.75 (95% CI: 0.64–0.87; mPFS 18.1 vs 14.1 months), and 0.71 (95% CI: 0.59–0.85; mPFS 36.1 vs 21.4 months) for complementary standard-risk patients. The HR for PFS with ixazomib- vs placebo-based therapy was 0.68 in patients with t(4;14) (95% CI: 0.48–0.96; mPFS 22.4 vs 13.2 months), and 0.77 for patients with amp1q21 (95% CI: 0.63–0.93; mPFS 18.8 vs 14.5 months). A PFS benefit was demonstrated with ixazomib- vs placebo-based therapy regardless of cytogenetic status, with greatest benefit observed in patients with t(4;14) and amp1q21.

## Introduction

Therapeutic strategies for multiple myeloma (MM) have greatly advanced in the last two decades following the introduction of novel agents such as immunomodulatory imide drugs, anti-CD38 antibodies and proteasome inhibitors (PIs) [1–3]. PIs have become a cornerstone of MM therapy [4], and their use in a continuous fashion or to achieve higher cumulative doses leads to improved long-term outcomes compared with fixed-duration therapy [5–7]. However, despite these improvements, outcomes of patients with MM vary based on a number of prognostic factors, including cytogenetic risk [8]. Indeed, the survival of patients with certain cytogenetic abnormalities has generally remained poor compared with patients with standard-risk cytogenetic abnormalities [3, 9–11]. Cytogenetic abnormalities associated with poorer prognosis in MM include deletion of chromosome 17p [del(17p)], translocation between chromosomes 4 and 14 [t(4;14)], translocation between chromosomes 14 and 16 [t(14;16)], and amplification of 1q21 (amp1q21) [3], and, aside from amp1q21, are included in the 2016 International Myeloma Working Group (IMWG) definition of high-risk cytogenetics [12].

There is a general consensus among clinicians that treatment with PIs benefit patients carrying these cytogenetic abnormalities [12]. This has been demonstrated for the oral PI ixazomib- vs placebo-based therapy in a number of phase 3 studies [13–17]. In TOURMALINE-MM1, relapsed, refractory, or relapsed and refractory multiple myeloma (RRMM) patients with high-risk cytogenetic abnormalities treated with ixazomib plus lenalidomide-dexamethasone (IRd) had similar progression-free survival (PFS) to those with standard-risk cytogenetics vs patients who received placebo-Rd [13], suggesting that addition of ixazomib to Rd may have abrogated the increased risk conferred by cytogenetic abnormalities [13]. Additionally, patients with high-risk and expanded high-risk cytogenetics treated with IRd demonstrated a treatment benefit vs placebo-Rd (overall survival [OS] hazard ratio [HR] < 1) in the final OS analysis of TOURMALINE-MM1 [14]. Similarly, TOURMALINE-MM2 showed that patients with newly diagnosed multiple myeloma (NDMM) with expanded high-risk cytogenetics treated with IRd had improved PFS compared with placebo-Rd [15]. In the maintenance setting, patients with high-risk cytogenetics who received ixazomib maintenance following autologous stem cell transplant (ASCT) in TOURMALINE-MM3 had improved PFS vs placebo [16], and in TOURMALINE-MM4, transplant-ineligible patients with expanded high-risk cytogenetics who received ixazomib as post-induction maintenance demonstrated a PFS benefit vs placebo [17].

Although identification of certain cytogenetic abnormalities has guided stratification of high-risk patients and helped to characterize prognosis, not all patients with high-risk cytogenetic abnormalities exhibit the same response to therapy, and the efficacy of available treatment options varies according to the presence of particular cytogenetic abnormalities [12]. Accordingly, treatment decisions should be based on individual cytogenetic data [18]. At present, clinical trials assessing response to MM therapy according to specific cytogenetic abnormalities are limited [19], and the IMWG has stated that the analysis of cytogenetic subgroups in trials comparing different treatments is an important goal [12]. We therefore conducted this analysis of four TOURMALINE studies (TOURMALINE- MM1, -MM2, -MM3 and -MM4) [13, 15–17] to evaluate the association between PFS and individual cytogenetic risk and to assess whether certain patients benefit more with ixazomib-based therapy, thus contributing to the evolution of precision medicine within MM.

## Methods

### Patients and study designs

This pooled analysis included data from four randomized, placebo-controlled phase 3 trials and two treatment interventions. The full methods for these trials have been published previously [13, 15–17]. In TOURMALINE-MM1 (NCT01564537), -MM2 (NCT01850524), -MM3 (NCT02181413), and -MM4 (NCT02312258), respectively, adult patients were enrolled in 147, 157, 167, and 187 sites across 26, 8, 30, and 34 countries.

Patients with RRMM (N = 722) were enrolled into TOURMALINE-MM1, and eligible patients in TOURMALINE-MM2 (N = 705) had a confirmed diagnosis of symptomatic MM according to IMWG criteria and were eligible for treatment with Rd but ineligible for ASCT. The study designs of TOURMALINE-MM1 and -MM2 were similar; patients were randomized 1:1 to receive oral ixazomib 4 mg or matching placebo on days 1, 8 and 15 of 28-day cycles. In addition, all patients received oral lenalidomide 25 mg on days 1–21 (10 mg for patients with creatine clearance ≤60 or ≤50 mL/minute, depending on local prescribing information) and oral dexamethasone 40 mg on days 1, 8, 15 and 22 [13, 15].

In TOURMALINE-MM3 and -MM4, 656 and 706 patients, respectively, with a confirmed diagnosis of symptomatic MM according to IMWG criteria were enrolled. In TOURMALINE-MM3, eligible patients had achieved ≥partial response (PR) after undergoing standard induction therapy followed by high-dose melphalan conditioning and single ASCT within 12 months of diagnosis. Eligible patients in TOURMALINE-MM4 were transplant-ineligible or unwilling to receive ASCT and had achieved ≥PR after 6–12 months of standard induction therapy. The TOURMALINE-MM3 and -MM4 studies were similarly designed; patients were randomized 3:2 to receive maintenance therapy with single-agent oral ixazomib 3 mg or matching placebo on days 1, 8 and 15 of 28-day cycles [16, 17].

The primary endpoint for all four studies was PFS, defined as the time from the date of randomization to the date of first documentation of disease progression or death from any cause, as assessed by an independent review committee. A key prespecified secondary endpoint in all four studies was PFS in high-risk cytogenetic patient groups characterized by specific cytogenetic abnormalities [13, 15–17].

The trials were conducted in accordance with the International Conference on Harmonization Guidelines for Good Clinical Practice and appropriate regulatory requirements. Local ethics committees or institutional review boards approved the protocols. All patients provided written informed consent.

### Pooled analysis patients

In this pooled analysis, evaluable patients were categorized into subgroups according to the presence of one or more cytogenetic abnormalities. Patients with del(17p) and/or t(4;14) and/or t(14;16) cytogenetic abnormalities were categorized as ‘high-risk’, and patients with ≥1 high-risk abnormality and/or amp1q21 were categorized as ‘expanded high-risk’. Complementary standard-risk cytogenetic subgroups were defined as the absence of these high-risk or expanded high-risk abnormalities in samples available for testing. If cytogenetic abnormalities were unknown, indeterminate, or missing, the patient was deemed as unclassifiable [13, 15–17].

### Cytogenetic assessments

In TOURMALINE-MM1 and -MM2, cytogenetic abnormalities were assessed by a central laboratory at screening (within 8 weeks of randomization in TOURMALINE-MM2) using fluorescence in situ hybridization (FISH) in enriched CD138+ plasma cells from bone marrow aspirates. The presence of del(17p), t(4;14), and t(14;16) were defined based on cut-offs of 5%, 3%, and 3% positive cells, respectively, based on the false-positive rates (or technical cut-offs) of the FISH probes used. Cut-off values of 3% (TOURMALINE-MM1) and 20% (TOURMALINE-MM2) were used for amp1q21, and ≥3 copies were considered positive for 1q21 (TOURMALINE-MM1 and -MM2) [13–15].

In TOURMALINE-MM3 and -MM4, cytogenetic assessments were performed locally using FISH or conventional karyotyping with locally defined thresholds for positivity. Result reports were interpreted centrally by a board-certified hematopathologist. In selected regions where cytogenetic evaluation at the time of diagnosis was not routinely conducted, pre-screening cytogenetic evaluation was performed where possible [16, 17]. Cytogenetic characteristics were documented at the time of disease diagnosis and were not reassessed during the course of the study (TOURMALINE-MM3) [16].

### Statistical analysis

Pooled data on progression events and deaths were used to generate Kaplan–Meier estimates for PFS for ixazomib- vs placebo-based therapy in the defined cytogenetic patient subgroups and for patients with ≥1 of the four individual cytogenetic abnormalities. Statistical comparisons of PFS in ixazomib- vs placebo-based therapy pooled treatment groups were based on unstratified HRs and descriptive log-rank *p* values [13, 15–17].

To control for potential confounding factors arising from differences between study designs and patient populations, a sensitivity analysis stratified by study was conducted by aggregating stratified HR from individual studies (weighted method to calculate overall HR). The pooled adjusted HR was derived from adjusting the determination of the overall HR applying the studies as covariate. The pooled weighted HR utilized the derived weights for each study, aggregated over the studies to obtain the pooled HR.

## Results

### Patients

Overall, 2247 patients in the pooled analysis population were evaluable for the presence or absence of del(17p), t(4;14), and t(14;16) cytogenetic abnormalities, of whom 497 (22%) were classified as high risk and 1750 (78%) were classified as standard risk (Table 1). Of these, 270 of 497 (54%) patients and 958 of 1750 (55%) patients received ixazomib-based therapy in the high-risk and complementary standard-risk subgroups, respectively (Table 1). Expanded high-risk cytogenetics (high-risk cytogenetic abnormalities and/or amp1q21) were evaluable in a total of 2098 patients (Table 1). Of these, 555 of 1143 (49%) patients receiving ixazomib-based therapy and 479 of 955 (50%) patients receiving placebo-based therapy had ≥1 high-risk cytogenetic abnormality and/or amp1q21, and were classified as expanded high risk (Table 1). Correspondingly, 588 (51%) and 476 (50%) of patients receiving ixazomib- and placebo-based therapy, respectively, were classified as standard risk (Table 1).

**Table 1:** Cytogenetic risk subgroups by study and treatment group.

	Evaluable for del(17p), t(4;14), and t(14;16) cytogenetic abnormalities *N* = 2247				Evaluable for del(17p), t(4;14), t(14;16), and amp1q21 cytogenetic abnormalities *N* = 2098			
Patients, *n* (%)	Ixazomib-based (*n* = 1228)		Placebo-based (*n* = 1019)		Ixazomib-based (*n* = 1143)		Placebo-based (*n* = 955)	
	High risk	Standard risk^a^	High risk	Standard risk^a^	Expanded high risk	Standard risk^a^	Expanded high risk	Standard risk^a^
TOURMALINE-MM1	75	200	62	216	155	122	154	126
TOURMALINE-MM2	60	231	63	234	134	164	146	153
TOURMALINE-MM3	61	252	54	152	116	154	88	89
TOURMALINE-MM4	74	275	48	190	150	148	91	108
Total	270 (22)	958 (78)	227 (22)	792 (78)	555 (49)	588 (51)	479 (50)	476 (50)

^a^Standard risk complement of (expanded) high risk (i.e., patients without any of the specified abnormalities).

### PFS analysis by cytogenetic risk subgroups

**Fig. 1:**
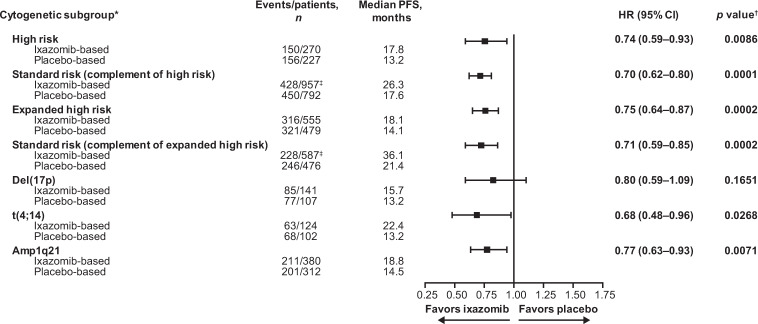
Forest plot of PFS by cytogenetic subgroup for patients receiving ixazomib- vs placebo-based therapy. High-risk subgroup: presence of one or more of del(17p), t(4;14) or t(14;16) cytogenetic abnormalities; expanded high-risk subgroup: presence of any of the above and/or amp1q21; complementary standard-risk subgroups: patients without any of the specified cytogenetic abnormalities listed above for high-risk and expanded high-risk. *Data for t(14;16) are not shown due to small patient numbers. ^†^Descriptive *p* values; analyses not powered for statistical testing. ^‡^1 patient was not included in the analysis due to inadequate sampling for cytogenetic assessment. CI confidence interval, HR hazard ratio, PFS progression-free survival.

**Fig. 2:**
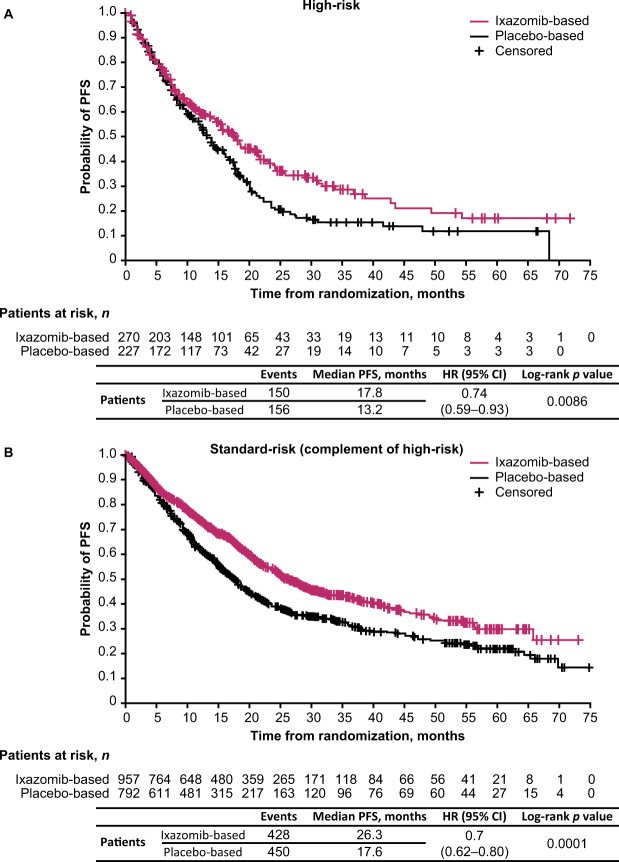
Kaplan–Meier estimates of PFS for patients with high- or standard-risk cytogenetic abnormalities receiving ixazomib- vs placebo-based therapy. **A** High-risk cytogenetic abnormalities, and **B** standard-risk (complement of high-risk) abnormalities. CI confidence interval, HR hazard ratio, PFS progression-free survival.

Median follow-up in the pooled analysis was 25.6 months (12.7, 54.6, 29.7, and 21.3 months, in TOURMALINE-MM1, TOURMALINE-MM2, TOURMALINE-MM3, and TOURMALINE-MM4, respectively). The HR for PFS with ixazomib- vs placebo-based therapy was 0.74 (95% confidence interval [CI]: 0.59–0.93; *p* = 0.0086) for patients in the high-risk subgroup, and the median PFS was 17.8 vs 13.2 months (Figs. 1 and 2A). For patients in the high-risk complementary standard-risk subgroup, the HR for PFS with ixazomib- vs placebo-based therapy was 0.70 (95% CI: 0.62–0.80; *p* = 0.0001; median PFS 26.3 vs 17.6 months) (Figs. 1 and 2B).

**Fig. 3:**
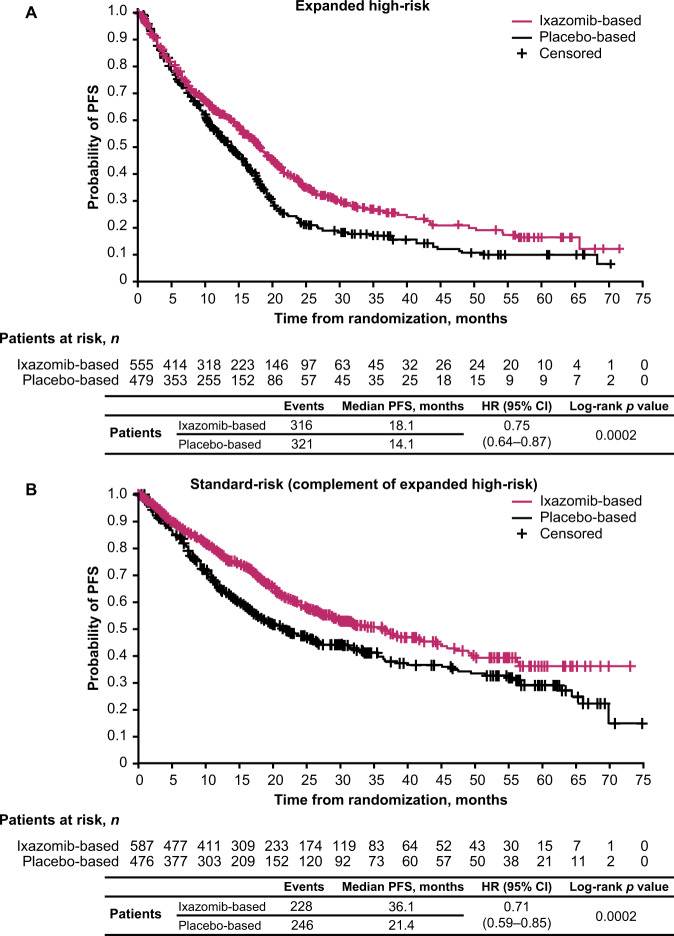
Kaplan–Meier estimates of PFS for patients with expanded high- or standard-risk cytogenetic abnormalities receiving ixazomib- vs placebo-based therapy. **A** Expanded high-risk cytogenetic abnormalities, and **B** standard-risk (complement of expanded high-risk) abnormalities. CI confidence interval, HR hazard ratio, PFS progression-free survival.

The HR for PFS with ixazomib- vs placebo-based therapy in the expanded high-risk subgroup was 0.75 (95% CI: 0.64–0.87; *p* = 0.0002). The median PFS was 18.1 months for patients receiving ixazomib-based therapy compared with 14.1 months for patients receiving the placebo-based regimen (Figs. 1 and 3A). For patients in the expanded high-risk complementary standard-risk subgroup, the HR for PFS with ixazomib- vs placebo-based therapy was 0.71 (95% CI: 0.59–0.85; *p* = 0.0002; median PFS 36.1 vs 21.4 months) (Figs. 1 and 3B).

When cytogenetic risk status was compared in patients receiving ixazomib-based therapy, median PFS for patients in the high-risk subgroup was 17.8 months, vs 26.3 months in the standard-risk subgroup (complement of high-risk) (HR: 0.65; 95% CI: 0.54–0.78; Supplementary Fig. 1A). For patients in the expanded high-risk subgroup, median PFS was 18.1 months, vs 36.1 months in the standard-risk subgroup (complement of expanded high-risk) (HR: 0.54; 95% CI: 0.46–0.64; Supplementary Fig. 1B).

### PFS analysis according to the presence of individual cytogenetic abnormalities

**Fig. 4:**
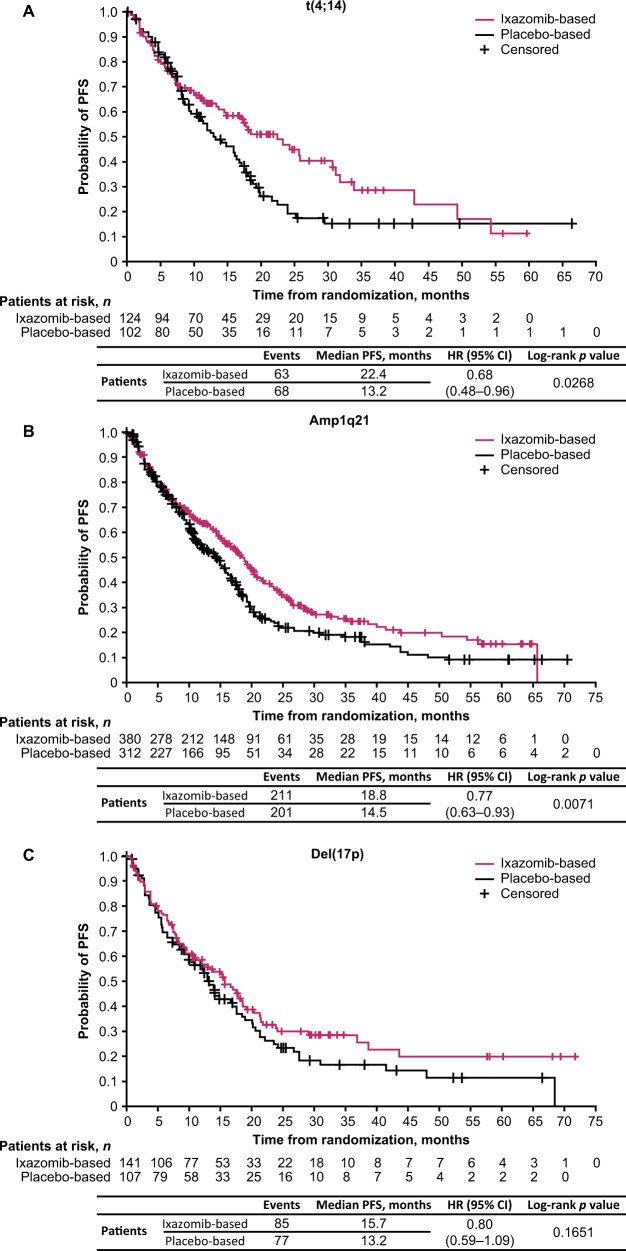
Kaplan–Meier estimates of PFS for patients with specific cytogenetic abnormalities receiving ixazomib- vs placebo-based therapy. **A** t(4;14) cytogenetic abnormality, **B** amp1q21 cytogenetic abnormality, and **C** del(17p) cytogenetic abnormality. CI confidence interval, HR hazard ratio, PFS progression-free survival.

In patients with t(4;14), the HR for PFS with ixazomib- vs placebo-based therapy was 0.68 (95% CI: 0.48–0.96; *p* = 0.0268; median 22.4 vs 13.2 months) (Figs. 1 and 4A). For patients with amp1q21, the HR for PFS with ixazomib- vs placebo-based therapy was 0.77 (95% CI: 0.63–0.93; *p* = 0.0071; median 18.8 vs 14.5 months) (Figs. 1 and 4B). For patients with del(17p), the HR for PFS with ixazomib- vs placebo-based therapy was 0.80 (95% CI: 0.59–1.09; *p* = 0.1651; median 15.7 vs 13.2 months) (Figs. 1 and 4C). To assess the impact of del(17p) on outcomes independent of treatment, we analyzed PFS according to the presence of del(17p) vs standard-risk cytogenetics in patients who received placebo-based therapy (Supplementary Fig. 2). The HR for PFS for patients with del(17p) vs standard-risk cytogenetics was 0.69 (95% CI: 0.54–0.88; *p* = 0.0023; median 17.6 vs 13.2 months). For the analysis in patients with t(14;16) there were only 11 events in 16 patients receiving ixazomib-based therapy and 14 events in 23 patients receiving placebo-based therapy; the median PFS was 11.4 and 15.9 months, respectively (HR 1.21; 95% CI: 0.55–2.69; *p* = 0.634) (Supplementary Fig. 3).

### Sensitivity analysis of PFS stratified by study

**Fig. 5:**
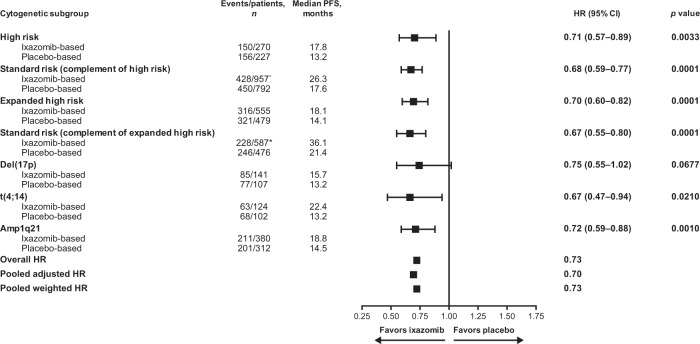
Forest plot of PFS with ixazomib- vs placebo-based therapy regardless of cytogenetic risk stratified by study. Sensitivity analysis conducted by aggregating stratified HR from the individual studies (weighted method to calculate overall HR), to accommodate for potential confounding factors arising from the differing study designs. Patients who were alive or who had not progressed at the data cut-off were censored on the day of last contact. High-risk subgroup: presence of one or more of del(17p), t(4;14) or t(14;16) cytogenetic abnormalities; expanded high-risk subgroup: presence of any of the above and/or amp1q21; complementary standard-risk subgroups: patients without any of the specified cytogenetic abnormalities listed above for high-risk and expanded high-risk. *1 patient was not included in the analysis due to inadequate sampling for cytogenetic assessment. CI confidence interval, HR hazard ratio, PFS progression-free survival.

A sensitivity analysis of PFS was performed; the HRs with ixazomib- vs placebo-based therapy were 0.71 (95% CI: 0.57–0.89; *p* = 0.0033) for patients with high-risk cytogenetic abnormalities and 0.68 (95% CI: 0.59–0.77; *p* = 0.0001) for patients in the complementary standard-risk subgroups. Similarly, the HRs for PFS with ixazomib- vs placebo-based therapy were 0.70 (95% CI: 0.60–0.82; *p* = 0.0001) for patients in the expanded high-risk subgroup, and 0.67 (95% CI: 0.55–0.80; *p* = 0.0001) for patients in the respective standard-risk subgroup (Fig. 5). In patients with del(17p), t(4;14), and amp1q21, the HRs for PFS with ixazomib- vs placebo-based therapy were 0.75, 0.67, and 0.72 (Fig. 5).

### PFS analysis by combination (non-maintenance) vs single-agent (maintenance) therapy

**Fig. 6:**
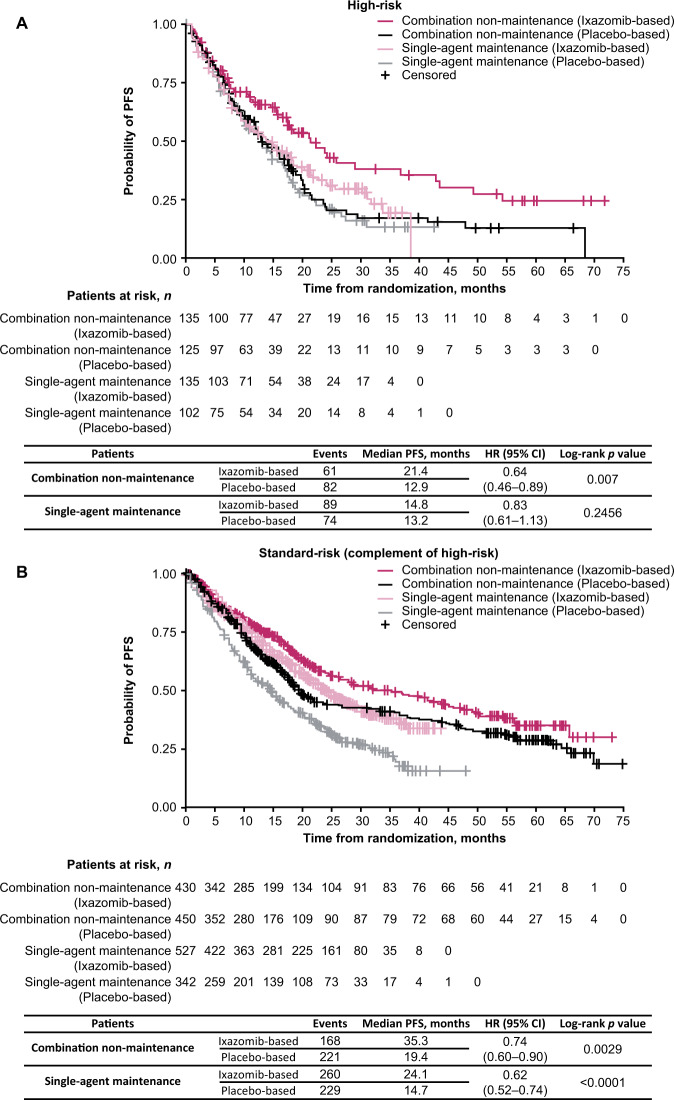
Kaplan–Meier estimates of PFS for patients receiving ixazomib- vs placebo-based therapy as single agent maintenance vs combination non-maintenance. **A** High-risk cytogenetic abnormalities, and **B** standard risk (complement of high-risk) abnormalities. CI confidence interval, HR hazard ratio, PFS progression-free survival.

Analyses of PFS to investigate whether ixazomib in combination with Rd (TOURMALINE-MM1 and TOURMALINE-MM2) improves outcomes for patients with high-risk cytogenetic abnormalities vs single-agent ixazomib (TOURMALINE-MM3 and TOURMALINE-MM4) are shown in Fig. 6.

In patients with high-risk cytogenetic abnormalities, the HR for PFS with ixazomib- vs placebo-based therapy in TOURMALINE-MM1 and TOURMALINE-MM2 (combination non-maintenance) was 0.64 (95% CI: 0.46–0.89; *p* = 0.007). In TOURMALINE-MM3 and TOURMALINE-MM4 (single-agent maintenance), the HR for PFS with ixazomib- vs placebo-based therapy was 0.83 (95% CI: 0.61–1.13; *p* = 0.2456) (Fig. 6A). The median PFS for patients receiving ixazomib as combination vs single-agent therapy was 21.4 vs 14.8 months. For patients receiving the placebo regimens, the median PFS was 12.9 vs 13.2 months for combination vs single-agent therapy (Fig. 6A).

For patients in the standard-risk subgroup, the HR for PFS with ixazomib- vs placebo-based in TOURMALINE-MM1 and TOURMALINE-MM2 (combination non-maintenance) was 0.74 (95% CI: 0.6–0.9; *p* = 0.0029); in TOURMALINE-MM3 and TOURMALINE-MM4 (single-agent maintenance) the HR for PFS with ixazomib- vs placebo-based therapy was 0.62 (95% CI: 0.52–0.74; *p* < 0.0001) (Fig. 6B). In the ixazomib treatment cohorts, the median PFS for patients was 35.3 vs 24.1 months for combination vs single-agent therapy, and for patients receiving the corresponding placebo regimen, median PFS was 19.4 vs 14.7 months, respectively (Fig. 6B).

## Discussion

This pooled analysis of four TOURMALINE phase 3 studies demonstrated a PFS benefit with ixazomib- vs placebo-based therapy regardless of the presence of specific adverse cytogenetic abnormalities in patients with MM. Ixazomib-based therapy prolonged median PFS by 4.6 and 4.0 months compared with placebo-based therapy for patients with high-risk and expanded high-risk cytogenetic abnormalities, respectively. Furthermore, a similar magnitude of benefit was found for ixazomib- vs placebo-based therapy for patients with high-risk (HR 0.74) and expanded high-risk (HR 0.75) cytogenetic abnormalities compared to the respective complementary standard-risk subgroups (HRs 0.70 and 0.71). Importantly, in subgroup analyses of specific cytogenetic abnormalities, a greater PFS benefit with ixazomib- vs placebo-based therapy was demonstrated for patients carrying t(4;14) and amp1q21 cytogenetic abnormalities. Overall, ixazomib combined with Rd or as a single-agent maintenance therapy did not abrogate the negative impact of high-risk cytogenetic abnormalities, consistent with previously reported findings with ixazomib in non-transplant NDMM patients (TOURMALINE-MM2 [15]), the maintenance setting (TOURMALINE-MM3 [16] and TOURMALINE-MM4 [17]), and smaller real-world studies [20, 21]. Additionally, in a recent study in NDMM patients, no significant improvement of PFS or OS was demonstrated with IRd maintenance vs Rd in patients with standard- or high-risk cytogenetics [22].

However, in TOURMALINE-MM1, median PFS data suggested that ixazomib in combination with Rd may improve prognosis for RRMM patients with high-risk cytogenetics by lengthening PFS to that similar to patients with standard-risk cytogenetics [13, 23]. Furthermore, greater OS benefit was observed in patients with high-risk and expanded high-risk cytogenetics treated with ixazomib [14]. The pooled analysis presented here included two studies of single-agent ixazomib (TOURMALINE-MM3 and TOURMALINE-MM4) and two studies where patients received IRd (TOURMALINE-MM1 and TOURMALINE-MM2). Thus, the triple-drug regimen may have influenced the ability of ixazomib to abrogate the negative impact of high-risk cytogenetics in this specific treatment setting, as suggested by the IMWG [12], but not all patients were exposed to the triple-drug regimen in the TOURMALINE program. We therefore conducted an analysis to assess the impact of single-agent ixazomib vs combination therapy, in both the high-risk and complementary standard-risk cohorts. Our findings show that in patients with high-risk cytogenetics abnormalities, there was a PFS benefit with ixazomib vs placebo in combination with Rd (HR 0.64) and the HR was lower than with single-agent ixazomib vs placebo (HR 0.83). In contrast, in patients with standard-risk cytogenetic abnormalities, a PFS benefit was observed with both combination and single-agent ixazomib, but the HR for PFS with IRd vs placebo-Rd (HR 0.74) was higher than the HR for single-agent ixazomib vs placebo (HR 0.62). These results support the findings from TOURMALINE-MM1 that ixazomib in combination with Rd is efficacious in patients with high-risk cytogenetic abnormalities and may improve the prognosis of these patients [13, 23].

Further analyses in the high-risk and expanded high-risk subgroups, according to specific cytogenetic abnormalities, demonstrated a PFS benefit with ixazomib- vs placebo-based therapy in patients with t(4;14) (HR 0.68), amp1q21 (HR 0.77), and del(17p) (HR 0.80). Ixazomib- vs placebo-based therapy increased median PFS for patients with t(4;14) and amp1q21 by 9.2 and 4.3 months, respectively. Ixazomib-based therapy prolonged median PFS by 2.5 months for patients with del(17p). The greater magnitude of PFS benefit seen for patients carrying t(4;14) and amp1q21 suggests that improved outcomes in the expanded high-risk subgroups were primarily driven by PFS differences in patients with these more frequent cytogenetic abnormalities. The number of patients in the t(14;16) subgroup were small and conclusions based on the relative contribution of this cytogenetic abnormality on PFS cannot be drawn.

This analysis supports the hypothesis that PI-based therapy is clinically relevant in the management of patients with MM who have high-risk cytogenetic abnormalities [12], and broadens the range of populations and treatment settings over which this benefit may apply. Other PI agents, including carfilzomib and bortezomib, have previously indicated a benefit in patients with high-risk cytogenetic abnormalities [24, 25], although outcomes with carfilzomib-Rd were still poorer than for patients with standard-risk cytogenetics [24], and other studies of short-course bortezomib-based therapy have reported limited benefit [26, 27]. This suggests prolonged PI-based therapy is important in this treatment setting. In the context of long-term therapy, carfilzomib and bortezomib are parenterally administered PIs [28, 29] vs the oral administration of ixazomib [30, 31]. Oral administration conveniently allows for less frequent hospital or clinic visits during continuous/prolonged PI treatment. Additionally, carfilzomib is associated with cardiac and renal complications, while peripheral neuropathy is a treatment emergent adverse event of clinical importance with bortezomib [32, 33]. The safety profile of ixazomib is well established [13, 15–17], and ixazomib-based therapy is feasible in patients with high-risk cytogenetics based on tolerability data from previous studies [13–17, 23].

The limitations of this pooled analysis include differences in eligibility criteria, patient populations, and length of follow-up among the four studies, as well as higher patient numbers in the standard-risk vs high-risk subgroups. Additionally, the copy number for chromosome 1 abnormalities evaluated in this analysis was not specified. A recent publication focusing on the lack of uniformity of categorizing patients with additional copies of 1q as high-risk highlights the importance of copy number of 1q as a driver of poor outcomes in MM [34]. As such, further study that includes the patients’ copy number of 1q is needed in order to determine the impact of this abnormality on treatment outcomes. Furthermore, data on measurable residual disease (MRD) were not collected for all four studies, thus an analysis of the relationship between MRD and cytogenetics cannot be conducted; however, a recent publication by Paiva et al. evaluates the implications of MRD in the TOURMALINE-MM3 and TOURMALINE-MM4 study populations [35]. Another limitation is the use of inconsistent cut-off values for defining the presence of high-risk cytogenetic abnormalities across the four studies in this analysis, limiting cross-trial comparisons. At present, there is no standardized FISH-positivity cut-off value for defining the presence of del(17p) [12, 23]. It is currently unclear what minimum percentage of del(17p)-positive cells is associated with a poor prognosis, or whether this varies depending on treatment used and stage of disease [12]. Previous MM studies involving cytogenetic assessments have used a range of different cut-off values for FISH testing, with some publications suggesting that a cut-off of >55% positive cells del(17p) is required to be deemed high risk [24, 36–39]. In TOURMALINE-MM1 and TOURMALINE-MM2, a cut-off of 5% positive cells was used for del(17p), and in TOURMALINE-MM3 and TOURMALINE-MM4 thresholds for positivity were defined locally. Given the differences in assessing del(17p) between the studies and that in our analyses we did not stipulate a cut-off for del(17p) to be considered high-risk compared with previous publications, we analyzed PFS in patients who received placebo-based therapy according to whether they were del(17p) positive vs standard-risk cytogenetics. The findings from this analysis suggest that the population of patients defined as del(17p) positive were high-risk as they had significantly lower PFS compared to those assessed as standard-risk. However, these results are based on pooled data with different levels of del(17p) positive cells present within individual patients, and the impact of this variability on these outcomes is unknown. Nonetheless, despite these limitations and the more conservative approach taken towards defining del(17p) positive patients, there was an overall trend in favor of ixazomib- vs placebo-based therapy.

In conclusion, ixazomib- vs placebo-based therapy demonstrated a PFS benefit in patients with MM regardless of the presence of specific adverse cytogenetic abnormalities. Ixazomib- vs placebo-based therapy displayed a similar magnitude of PFS benefit in patients with high-risk and expanded high-risk cytogenetic abnormalities compared to the respective complementary standard-risk subgroups. Our results suggest that ixazomib- vs placebo-based therapy may provide a greater PFS benefit for patients carrying t(4;14) and amp1q21. Additionally, we demonstrate a PFS benefit for ixazomib in combination with Rd vs single-agent ixazomib in patients with high-risk MM. In line with previously reported findings, ixazomib combined with Rd or as single-agent maintenance therapy did not abrogate the negative impact of high-risk cytogenetic abnormalities. However, differences in eligibility criteria and patient populations across the four studies may have contributed to difficulties interpreting the data. Overall, the findings from this pooled analysis indicate that ixazomib-based therapy may be a viable treatment option for patients with MM who have high-risk and expanded high-risk cytogenetic abnormalities.

## Supplementary information

Supplemental material

## Data Availability

The datasets, including the redacted study protocol, redacted statistical analysis plan, and individual participants’ data supporting the results reported in this article, will be made available within 3 months from initial request to researchers who provide a methodologically sound proposal. The data will be provided after its de-identification, in compliance with applicable privacy laws, data protection, and requirements for consent and anonymization.
