# Supplementary material for: A pooled analysis of outcomes according to cytogenetic abnormalities in patients receiving ixazomib- vs placebo-based therapy for multiple myeloma
Source: Blood Cancer J. 2023 Jan 12;13(1):14. doi: 10.1038/s41408-022-00768-5 (PMC9834310; doi:10.1038/s41408-022-00768-5)
Supplement: Supplementary file 1 — Supplemental material [file 41408_2022_768_MOESM1_ESM.pdf]

SUPPLEMENTARY INFORMATION

Supplementary figures

Supplementary Fig. 1 Kaplan-Meier estimates of PFS for patients receiving ixazomib with (A) high-risk cytogenetic abnormalities, and B) expanded high-risk cytogenetic abnormalities, compared with standard-risk abnormalities.

A

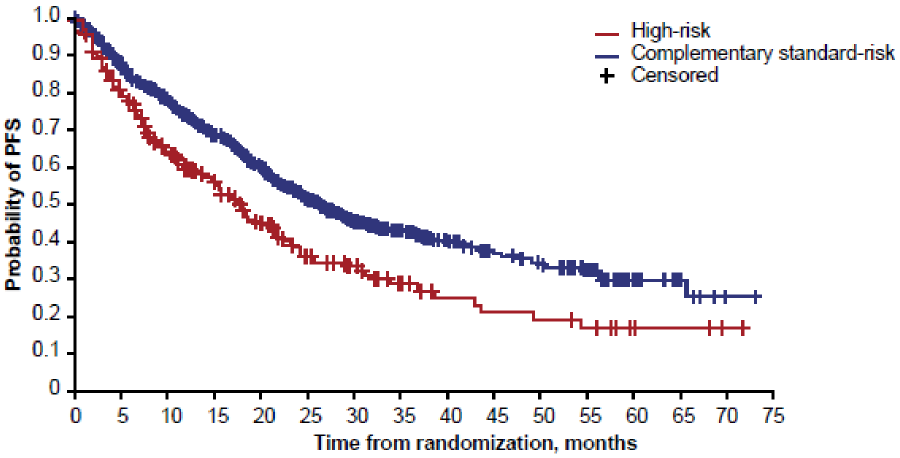

Patients at risk, *n*

High-risk	270	203	148	101	65	43	33	19	13	11	10	8	4	3	1	0
Complementary standard-risk	957	764	648	480	359	265	171	118	84	66	56	41	21	8	1	0

		Events	Median PFS, months	HR (95% CI)
Patients	High-risk	150	17.8	0.65
	Complementary standard-risk	428	26.3	(0.54–0.78)

B

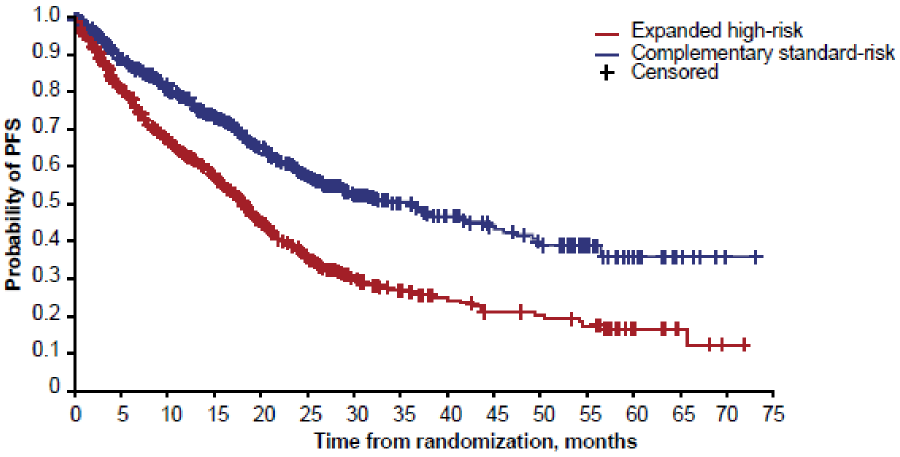

Patients at risk, *n*

Expanded high-risk	555	414	318	223	146	97	63	45	32	26	24	20	10	4	1	0
Complementary standard-risk	587	477	411	309	233	174	119	83	64	52	43	30	15	7	1	0

		Events	Median PFS, months	HR (95% CI)
Patients	Expanded high-risk	316	18.1	0.54
	Complementary standard-risk	228	36.1	(0.46–0.64)

CI confidence interval; *HR* hazard ratio; *PFS* progression-free survival.

Supplementary Fig. 2 Kaplan-Meier estimates of PFS for patients with del(17p) vs standard-risk cytogenetic abnormalities receiving placebo-based therapy.

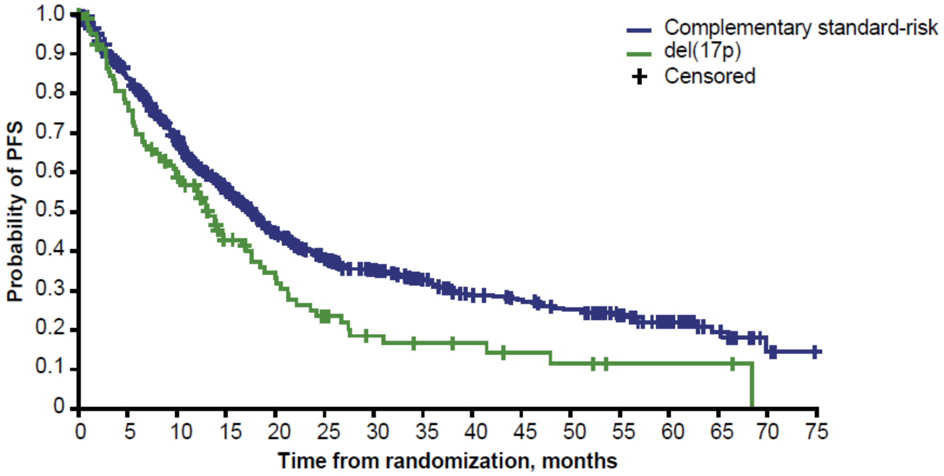

Patients at risk, *n*

Complementary standard-risk	792	611	481	315	217	163	120	96	76	69	60	44	27	15	4	0
del(17p)	107	79	58	33	25	16	10	8	7	5	4	2	2	2	0	

		Events	Median PFS, months	HR (95% CI)	Log-rank *p* value
**Patients**	Complementary standard-risk	77	17.6	0.69 (0.54–0.88)	0.0023
	del(17p)	450	13.2		

CI confidence interval; *HR* hazard ratio; *PFS* progression-free survival.

Supplementary Fig. 3 Kaplan-Meier estimates of PFS for patients with t(14;16) cytogenetic abnormality receiving ixazomib- vs placebo-based therapy.

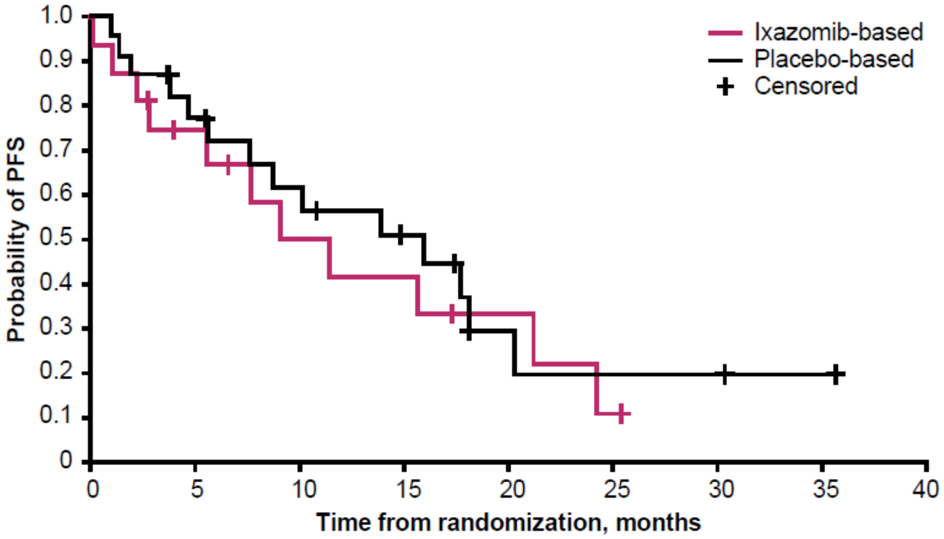

Patients at risk, *n*

	0	5	10	15	20	25	30	35	40
Ixazomib-based	16	10	6	5	3	1	0		
Placebo-based	23	16	12	8	3	2	2	1	0

		Events	Median PFS, months	HR (95% CI)	Log-rank *p* value
**Patients**	Ixazomib-based	11	11.4	1.21 (0.55–2.69)	0.634
	Placebo-based	14	15.9		

CI confidence interval; *HR* hazard ratio; *PFS* progression-free survival.
